# Supplementary material for: Seminoma and Embryonal Carcinoma Footprints Identified by Analysis of Integrated Genome-Wide Epigenetic and Expression Profiles of Germ Cell Cancer Cell Lines
Source: PLoS One. 2014 Jun 2;9(6):e98330. doi: 10.1371/journal.pone.0098330 (PMC4041891; doi:10.1371/journal.pone.0098330)
Supplement: File S1 — ZIP file containing DMRforPairs output for significant regions. Please start from the html files. (ZIP) [file pone.0098330.s008.zip › figures/5927.pdf]

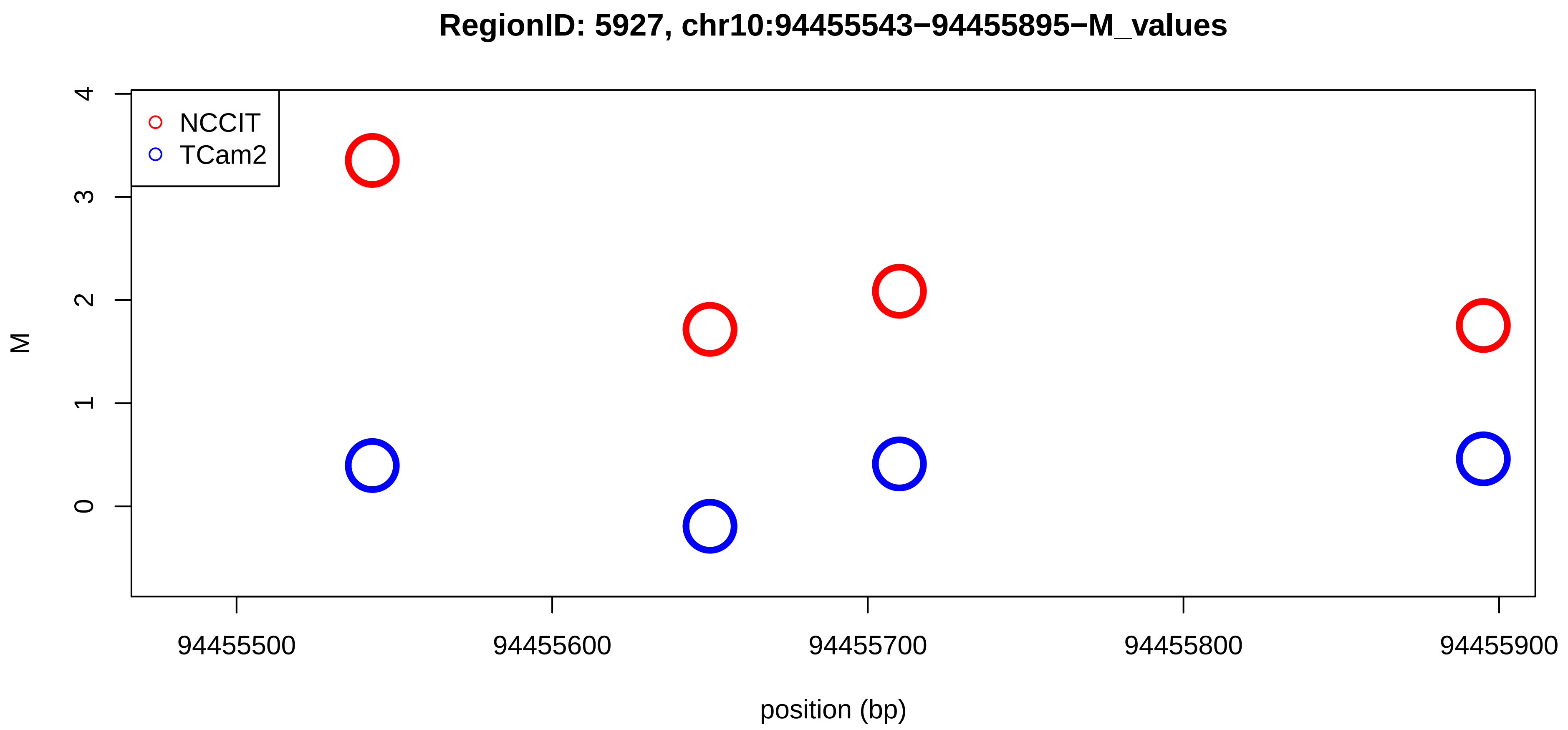

RegionID: 5927, chr10:94455543−94455895−M_values
NCCIT
TCam2
M
4
3
2
1
0
94455500
94455600
94455700
94455800
94455900
position (bp)

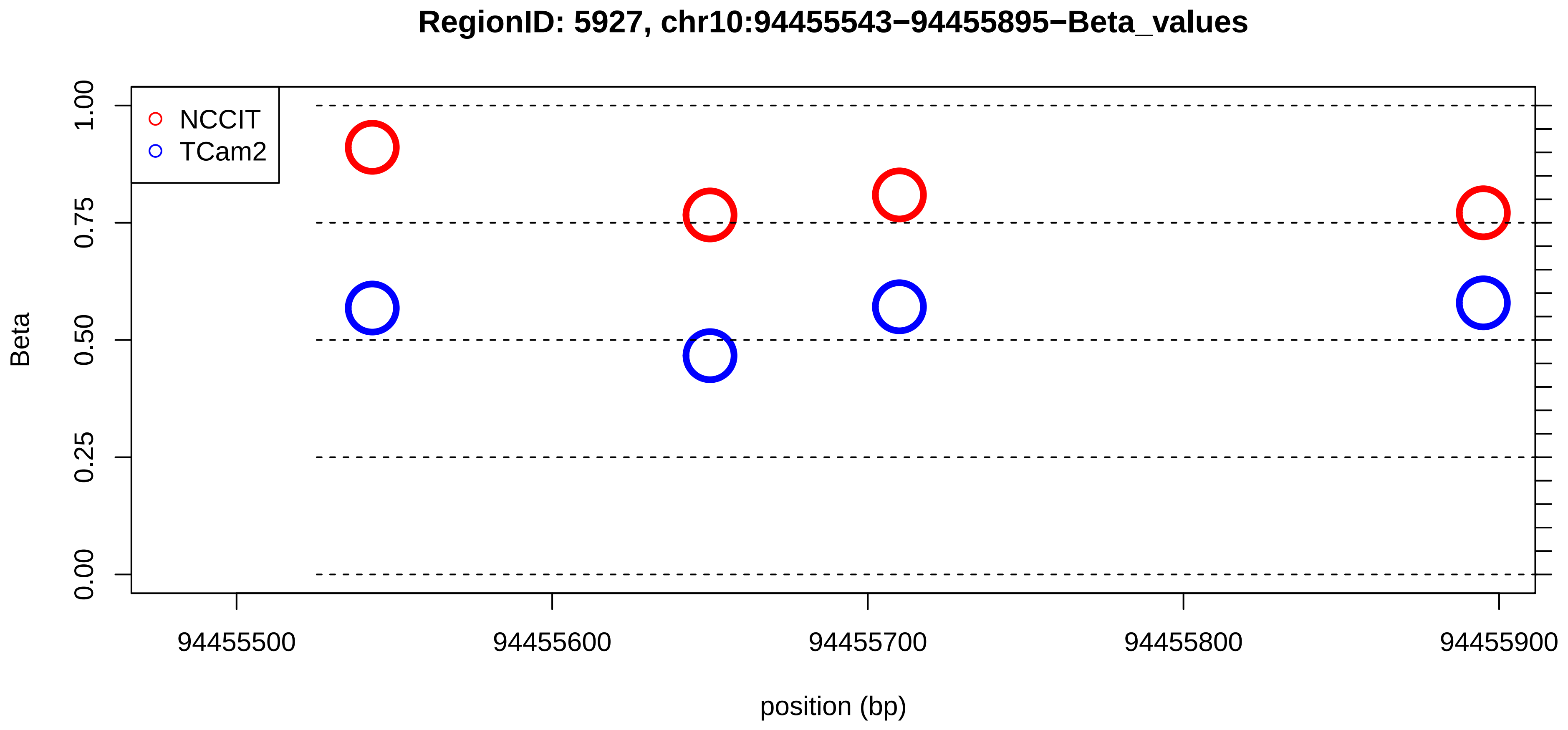

RegionID: 5927, chr10:94455543−94455895−Beta_values
NCCIT
TCam2
Beta
1.00
0.75
0.50
0.25
0.00
94455500
94455600
94455700
94455800
94455900
position (bp)
